# Exploring women’s preferences for birth settings in England: A discrete choice experiment

**DOI:** 10.1371/journal.pone.0215098

**Published:** 2019-04-11

**Authors:** Benjamin Rupert Fletcher, Rachel Rowe, Jennifer Hollowell, Miranda Scanlon, Lisa Hinton, Oliver Rivero-Arias

**Affiliations:** 1 Policy Research Unit in Maternal Health and Care, National Perinatal Epidemiology Unit, Nuffield Department of Population Health, University of Oxford, Oxford, United Kingdom; 2 Nuffield Department of Primary Care Health Sciences, University of Oxford, Oxford, United Kingdom; 3 BirthChoiceUK, London, United Kingdom; University of Brighton, UNITED KINGDOM

## Abstract

**Objective:**

To explore pregnant women’s preferences for birth setting in England.

**Design:**

Labelled discrete choice experiment (DCE).

**Setting:**

Online survey.

**Sample:**

Pregnant women recruited through social media and an online panel.

**Methods:**

We developed a DCE to assess women’s preferences for four hypothetical birth settings based on seven attributes: reputation, continuity of care, distance from home, time to see a doctor, partner able to stay overnight, chance of straightforward birth and safety for baby. We used a mixed logit model, with setting modelled as an alternative-specific constant, and conducted a scenario analysis to evaluate the impact of changes in attribute levels on uptake of birth settings.

**Main outcome measures:**

Women’s preferences for birth setting.

**Results:**

257 pregnant women completed the DCE. All birth setting attributes, except ‘time to see doctor’, were significant in women’s choice (p<0.05). There was significant heterogeneity in preferences for some attributes. Changes to levels for ‘safety for the baby’ and ‘partner able to stay overnight’ were associated with larger changes from baseline uptake of birth setting. If the preferences identified were translated into the real-world context up to a third of those who reported planning birth in an obstetric unit might choose a midwifery unit assuming universal access to all settings, and knowledge of the differences between settings.

**Conclusions:**

We found that ‘safety for the baby’, ‘chance of a straightforward birth’ and ‘can the woman’s partner stay overnight following birth’ were particularly important in women’s preferences for hypothetical birth setting. If all birth settings were available to women and they were aware of the differences between them, it is likely that more low risk women who currently plan birth in OUs might choose a midwifery unit.

## Introduction

Current guidelines in England state that women at low risk of complications should be offered a choice of birth setting and that available options should include support for home birth, freestanding midwifery units (FMUs), alongside midwifery units (AMUs), and labour wards/obstetric units (OUs).[1] Evidence from the 2011 Birthplace cohort study was used to inform this recommendation.[2] That study found for healthy women with straightforward pregnancies, planned birth was similarly safe for babies across settings, with the exception of planned birth at home for nulliparous women, and that planning birth at home or in a midwifery unit (MU) was associated with lower rates of intervention (augmentation, epidural, instrumental birth, caesarean).

However, not *all* women have access to *all* options. A study found that in 2008 only 4.7% of women in England had access to all four settings.[3] Since then coverage has increased, with the number of AMUs doubling in England since 2010, and the number of FMUs increasing slightly from 58 to 61.[4] However, there has also been significant ‘churn’ with 30 units opening and 21 permanently closing.[5] In 2013, 79% of women lived within 30 minutes’ drive of both an OU and a midwifery unit.[6]

Despite this apparent increase in the provision of, and support for, different birth settings, most women still give birth in obstetric units.[6] In 2015 around 14% of women gave birth in MUs,[4] and 2% gave birth at home.[7] Given the availability of options and policy focus, it is perhaps surprising that in a 2014 national survey a third of women reported being aware of only one option of place of birth.[8] In 2017 the Care Quality Commission reported that 42% of women were offered a choice of giving birth in a MU and 38% were offered the option of a home birth.[9]

There have been a number of studies investigating birth choice, carried out, in the main, in countries that actively promote birth choice for women at low-risk of complications, including the Netherlands[10, 11], Denmark[12], Australia[13], New Zealand[14] and Canada[15]. In the UK there is little current or high quality evidence about the factors that influence women’s choice of birth setting or what women value when making decisions about place of birth.[16, 17]

Given the discrepancy between the services that are available to women and where women actually give birth, the aims of this study were: to better understand what is important to women when making decisions about where to give birth; and to identify those service attributes that women prioritise over others. This study was part of a broader project to generate evidence to inform decisions about the commissioning and delivery of maternity services that support choice.[16–18] This study focuses on women’s stated preferences for birth setting, which is only one contributing factor in determining where women give birth. Other factors include the birth settings that are available to women in their local area, whether these different settings are presented to women as options, the extent to which women have sufficient information to enable them to make an informed decision, and whether complications arise during pregnancy or labour which lead to changes in planned or actual birth setting. While these factors are important in determining where women give birth, they are all beyond the scope of this study.

## Methods

### Discrete choice experiments

Decisions in health often extend beyond what is most effective, to include other considerations such as costs, convenience, availability, ease of use and potential risks.[19] A number of these are susceptible to the judgements of health service users, and can therefore be important in the success or failure of different options, or the level of uptake of a service. Discrete choice experiments (DCEs) are a well-established method used to understand the value individuals place on health and healthcare. DCEs elicit the strength of preferences for different ‘attributes’ or ‘characteristics’ of a number of alternatives describing health care intervention or services (e.g. home birth, FMU, AMU, OU).[20] Each attribute has a number of different ‘levels’, for example, in our DCE the attribute ‘time to see doctor’ had five levels ranging from ‘0 to 10 minutes’, to ‘greater than 60 minutes’. In a DCE participants are presented with a series of choice sets. In each set the participant is presented with usually two hypothetical alternatives, each with differing levels of the attributes being investigated, and is asked to indicate which of the two options they prefer.[21]

In this study, in order to inform future service provision, we were interested in the factors that are important to women when making decisions about place of birth. To explore this issue we have two options: we could ask women “What did you do?” or “What would you do?” When investigating the former question, known as ‘revealed preference’, we could gather information about what services are available, what information is available to women when making decisions about birth choice, and what birth settings women choose. This can tell us much about real-world decision making, but women’s the choices are constrained by what options are available to them, and what information they have been provided with. Addressing the second question of “What would you do?” requires a ‘stated preference’ approach, such as a DCE. By investigating how women would make choices about birth setting given full availability of all options, incorporating service attributes that may not be currently available to all women, this allows us to establish what women value irrespective of what is actually on offer. A DCE helps us quantify the value that women attach to each birth setting and predict demand for future services. The extent to which the results of stated preference studies (including DCEs) translate into real-world settings (i.e. external validity) is an emerging research area with some promising findings.[22]

### Identifying attributes and levels

We used a number of sources of information to inform the development and refinement of birth setting attributes. An initial candidate set of attributes and levels was developed using two systematic reviews [16, 17], and primary research (a series of nationwide focus groups) conducted for this project. [18] This initial set included six attributes: ‘reputation’ (whether friends/family would recommend a setting); ‘continuity of care’ (the extent to which women know the midwife who looks after them during labour and birth); ‘distance from home’ expressed as travel time; ‘time to see a doctor’ (e.g. should complications arise during labour or birth); ‘chance of straightforward birth without intervention’; and ‘safety for the baby’.

We discussed the number of attributes, the definition of each attribute, and refinement of the language used for attributes and levels with a stakeholder feedback group comprising representatives from a range of organisations/groups with an interest in birth setting and choice (see acknowledgments). After this stakeholder meeting, ten pregnant women (PPI representatives) were invited to pilot the questionnaire using a “think aloud” approach whereby participants were asked to give a commentary on their thoughts while completing the questionnaire, and recall their thoughts immediately following completion of the task.[23] This helped assess whether participants were engaged with the DCE, whether they took all of the information presented to them into account when stating a preference, and also informed our decision to present 9–16 choice questions. As a result of the “think aloud” task we decided to create an online video to complement the information already provided describing the study design and attributes in text and added an additional attribute about whether a partner can stay overnight following the birth. Thus the final DCE included seven attributes, presented in Table 1 with their associated levels. Information presented to women about birth settings is available in Appendix A in S1 File.

**Table 1:** Description of alternatives, attributes and levels.

**Birth setting alternatives**	[0] Home
	[1] Freestanding midwifery unit
	[2] Alongside midwifery unit
	[3] Labour ward
**Attribute**	**Levels**
Reputation	[0] Your friends/family have had a **poor experience** of this option and **would not recommend**
	[1] Your friends family have **no previous experience** of this option
	[2]Your friends/family have a **good experience** of this option and **would recommend**
Continuity of care	[0] You get to know your midwife during your pregnancy and where possible they look after you throughout labour and birth.
	[1] You meet a team of 4–6 midwives during pregnancy, one of whom looks after you throughout labour and birth
	[2] You meet the midwife for the first time during labour (e.g. on arrival at the unit), and they look after your throughout labour and birth.
	[3] You meet a midwife for the first time during labour. They will look after you during labour and birth, but if the unit is busy they may look after other women in labour at the same time.
Distance from home	[0] 0 to 15 minutes
	[1] 15 to 30 minutes
	[2] 30 to 60 minutes
	[3] More than 60 minutes
Time to see a doctor	[0] 0 to 10 minutes
	[1] 10 to 20 minutes
	[2] 20 to 40 minutes
	[3] 40 to 60 minutes
	[4] More than 60 minutes
Can your partner stay overnight after the birth of your baby	[0] Your partner **cannot stay** with you overnight
	[1] Your partner **can stay** with you on a **ward shared with others**
	[2] Your partner **can stay** with you in a **room not shared with others**
Chance of straightforward birth	[0] **5 out of 10** women have **straightforward birth**, 5 out of 10 have intervention
	[1] **6 out of 10** women have **straightforward birth**, 4 out of 10 have intervention
	[2] **7 out of 10** women have **straightforward birth**, 3 out of 10 have intervention
Safety for baby	[0] **Slightly worse than average** (10 out of 1,000 have a poor outcome for baby, 990 of 1,000 births baby is born healthy)
	[1] **Average** (4 out of 1,000 births have poor outcome for baby, 996 of 1,000 births baby is born healthy)
	[2] **Slightly better than average** (2 out of 1,000 births have a poor outcome for baby, 998 of 1,000 births baby is born healthy)

### Study design and survey

The DCE presented women with choice sets of hypothetical birth settings that differed according to the levels assigned to the seven attributes of interest (example of DCE question available in Appendix C in S1 File). Rather than present women with four ‘settings’ for each set we decided to present two alternatives in each choice set to reduce the burden on participants. The final number of potential choice sets was identified using a D-optimal design approach allowing estimation of main-effects to generate the final choice set using Ngene (ChoiceMetrics version 1.1.2, 2012). A number of restrictions were included in the design, for example, the level for the attribute “distance from home” was always “0” when the attribute “setting” was “home”. The final design resulted in 60 choice sets divided into four blocks, and had a D-efficiency score of 96.9%. See Appendix B in S1 File for NGene syntax, attribute restrictions, and final design.

The DCE survey was developed using the open source survey tool LimeSurvey (www.limesurvey.org), and administered online (a screen shot of a DCE question as it appeared on-line is available in Appendix C in S1 File). The online survey incorporating the DCE comprised a question about consent, followed by questions about the woman’s pregnancy (parity and risk of complications). These were followed by the DCE questions, a question about whether women had already made a decision about where to give birth, and if so where, and a number of demographic questions: age, region, ethnicity, education, and employment. Women were also asked to rate the three most and least important attributes when making decisions in the DCE. After completion of the consent and pregnancy questions, participants were randomised to one of the four blocks of 15 choice sets. The order of the questions within each block was also randomised. The survey was piloted by members of the research team and with PPI representatives (some of the participants who had taken part in the ‘think aloud’) to test the format and ordering of the questions, and to provide an estimate of the time taken to complete. The online DCE survey was optimised to function with desktops, laptops and tablets but not mobile phones.

### Data collection and participants

We invited pregnant women over the age of 18 in England to take part in the survey. The study was first advertised on social media (Twitter) on 17^th^ January 2018. After slow recruitment and poor completion rates we engaged an online panel company to help achieve the target sample size. The company conducted a brief survey of their panel to explore access to pregnant women with interest in completing our survey. Participating women identified through the online panel received reward points that could be redeemed for vouchers or goods on completion of valid surveys. Sampling through the on-line panel began on 9^th^ March 2018 and the survey was closed on 23^rd^ March 2018.

### Sample size consideration

Sample size was calculated using the “rule of thumb” method proposed by Johnson and Orme for DCEs without prior information (details in Appendix D in S1 File).[24, 25] For this study it was estimated that at least 168 participants would be required, and we therefore aimed to sample at least 200 women.

### Data analysis

Participant demographics were summarised using descriptive statistics. Responses to the DCE tasks were modelled assuming the random utility model (RUM), whereby the latent utility *U*_*in*_ of an alternative *i* in a choice set has two separate parts: (i) a systematic (explainable) component *V*_*in*_ specified as a function of the attributes of the alternatives and (ii) a random (unexplainable) component *ε*_*in*_ representing unmeasured variation in responses. The relationship between the systematic and the unmeasured component is additive such as *U*_*in*_ = *V*_*in*_ + *ε*_*in*_. According to utility maximisation, woman *n* selected alternative *i* if the alternative maximised her utility (satisfaction) among all alternatives in the choice set.[24] *V*_*in*_ was assumed to be linear and additive function of each settings attributes and levels as follows:

$$V_{in}=\beta_{i0}+\beta_{1}Reputation+\beta_{2}Continuity+\beta_{3}Distance\;from\;home+\beta_{4}Time\;to\;see\;doctor+\beta_{5}Partner\;stay+\beta_{6}Straightforward\;birh+\beta_{7}Safety\;for\;Baby$$

with *β*_*i*0_ indicating alternative-specific constants (ASCs) capturing women’s preferences for a particular ‘setting’. Therefore, our model included three ASCs (FMU, AMU and OU) with home birth as reference and all attributes were available in all four settings. Significant ASCs coefficients indicate that there were elements of the decision not captured by the list of attributes in the DCE. All remaining variables were categorical and the sign of the coefficients denoted whether a move from the reference/base category for each variable resulted in an increase or decrease in overall utility (satisfaction).

We did not attempt to include women’s characteristics in the explainable component *V*_*in*_ but we estimated a random parameter mixed logit to account for random variation across women.[26] Random variation in this context means heterogeneity that cannot be explained using women’s characteristics collected as part of our survey. The presence of heterogeneity in our sample is represented by the estimated standard deviations associated to each model parameter. Significant standard deviations indicate the presence of random heterogeneity.

All parameters (including the ASCs) were assumed to be normally distributed and we employed 5,000 random draws. We used Stata's modified Newton-Raphson algorithm for the maximum likelihood estimation and used the cluster option at the participant level to recognise that everyone completed 15 choice sets. A significance level of 5% was selected to determine statistically significant coefficients and standard deviations.

### Post estimation scenario analysis

We used the results of the random parameter mixed logit to conduct a post-estimation scenario analysis to investigate what the predicted uptake of birth settings would be if all were available to women, i.e. what is the value that women place on each birth setting. The predicted probabilities that women would choose each setting was estimated assuming participants had access to all four settings and all else being equal (in this case all other attributes set to the baseline category). The impact single level changes have on overall predicted uptake (e.g. we estimated how the predicted uptake across settings changed if the chance of straightforward birth at home increased from 50% to 60%) was also assessed.[27] We evaluated the impact of level changes in overall predicted uptake for the seven attributes in the DCE, and present these results using the absolute change in predicted uptake from baseline and associated 95% confidence intervals.

All analyses were conducted using Stata 14 (StataCorp. 2015. Stata Statistical Software: Release 14. College Station, TX: StataCorp LP).

### Details of ethics approval

Ethical approval for this study was granted by the University of Oxford Medical Sciences Interdivisional Research Ethics Committee on December 1^st^ 2017 (IDREC ref: R54678/RE001)

## Results

### Respondent characteristics

The online survey was available for 51 days between 17^th^ January and 9^th^ March 2018, and was accessed by 603 women, 102 from advertising through social media, and 501 from the on-line panel. Overall 87% (523/603) of those who accessed the survey completed at least one question and 49% of these (257/523) completed the whole survey, representing 43% of all those who accessed the survey. Most of those who started the DCE section completed the whole survey (95%, 257/271).

Participant demographics are presented in Table 2 along with national data available from the Office for National Statistics. Most women were white (81%), educated to at least undergraduate degree level (60%), and currently in paid work (62%). For most women this was not their first pregnancy (63%), and most reported being at low risk of complications arising during pregnancy and birth (71%). Women were represented geographically in similar proportions to national data.

**Table 2:** Participant characteristics.

	Participants completing the DCE survey (n = 257)	National data specific to pregnant women/mothers where available*
**Age**		
(Mean, sd)	29.9 (6.4)	30.4
(Median, IQR)	30 (20–44)	
**Parity (n, %)**		
Nulliparous	95 (37%)	41%
Multiparous	162 (63%)	59%
**Risk of complications (n, %)**		
No	182 (71%)	
Yes	65 (25%)	
Don’t know	10 (4%)	
**Region (n, %)**		
East	16 (6%)	11%
East Midlands	19 (7%)	8%
Greater London	71 (28%)	20%
North East	14 (5%)	4%
North West	29 (11%)	13%
South East	37 (14%)	15%
South West	23 (9%)	9%
West Midlands	24 (9%)	11%
Yorkshire and Humber	18 (7%)	10%
No answer	6 (2%)	
**Ethnicity (n, %)**		
White	207 (81%)	72%
Asian/Asian British	27 (11%)	9%
Black/African/Caribbean/Black British	5 (2%)	4%
Mixed/Multiple Ethnic groups	10 (4%)	
Other	3 (1%)	12%
Prefer not to answer	2 (1%)	
No answer	3 (1%)	4%
**Education (n, %)**		
No qualifications	1 (0.5%)	
O levels, GCESs, BTEC, NVW, or similar	40 (16%)	
A levels or technical qualifications	58 (23%)	
Degree level	102 (40%)	
Postgraduate degree	52 (20%)	
No answer	4 (2%)	
**Employment (n, %)**		
Unemployed	11 (4%)	
In education	12 (5%)	
In paid work	159 (62%)	
Looking after family/home/dependents	28 (11%)	
On maternity leave	38 (15%)	
Unable to work due to disability	2 (1%)	
Prefer not to say	3 (1%)	
No answer	4 (2%)	

*Office for National Statistics Statistical Bulletin: Births in England and Wales (2016/2017)

Participants completed the survey on average in just over nine minutes (mean 545 seconds, s.d. 457).

### Birth setting options available to women

Most women reported that an OU was available to them in their local area (86.8%, 223/257), and that there was support for home birth (73.9%, 190/257). Fewer participants were aware of either an AMU (53.3%, 137/257) or FMU (38.9%, 100/257) in their local area. The majority of women reported that they had already made a decision about where to give birth (199/257, 77.4%), with most choosing an OU (62.3%, 124/199), followed by AMU (19.1%, 38/199), home (12.6, 25/199) and FMU (6%, 12/199).

We asked participants to choose the three most important and three least important attribute when choosing where to give birth, and full results are available in Table A in S1 File. Each attribute was important for some women when making decisions, and less so for others, highlighting the complexity of the decision making process. For example, ‘safety for baby’ was selected as one of the three most important attributes by 46.3% of women and as one of the three least important by 22.2%.

When asked which sources of information were useful when making their decision about where to give birth, the most popular options were: midwife (68%, 176/257), online (48%, 124/257) and GP (39%, 101/257) (full results in Table B in S1 File).

### Women’s preferences for birth settings

Results from the random parameter mixed logit model are presented in Table 3. The ASCs for FMU, AMU and OU were positive and statistically significant, indicating that these birth settings were preferred to home birth. The significant coefficients also indicate that the ASCs for each setting captured elements of the decision not included in the list of attributes in the DCE. All but one of the attributes (time to see doctor) had a least one significant coefficient, indicating that they were all important when selecting a particular birth setting scenario. Settings that were recommended by family/friends were preferred over those where family/friends had a poor experience or those where family/friends had no previous experience. For continuity of care, having the same midwife throughout pregnancy and birth (reference category) was preferred over meeting a midwife for the first time during labour, although the other two levels of this attribute were not significantly different to the reference category. The coefficients for distance from home were negative and significant, showing that all were less attractive than the reference category (0–15 minutes) indicating that in general, women preferred a birth setting closer to home. Women had a strong preference for their partner to stay overnight with them, either on a ward shared with others, or in a room not shared with others. Women preferred birth settings where the chance of a straightforward birth without intervention was highest. Safety for baby was a significant driver of preferences, with women consistently selecting scenarios where safety was average or slightly better than average.

**Table 3:** Parameter coefficients from the random parameter mixed logit model.

**PARAMETERS**			
Variable	Coefficient	Confidence interval	p-value
**Alternative-specific constants (ASCs)** [Home reference ASC]	[0]		
FMU	0.461	0.129 to 0.792	0.006
AMU	0.682	0.322 to 1.043	0.000
Labour ward	0.907	0.548 to 1.266	0.000
**Reputation** [Poor experience]	[0]		
No previous experience	0.198	0.025 to 0.372	0.025
Good experience	0.403	0.238 to 0.568	0.000
**Continuity of care** [Same midwife throughout]	[0]		
One of a team of midwives throughout	-0.131	-0.321 to 0.059	0.177
Meet midwife for first time during labour, only look after you	-0.208	-0.377 to -0.039	0.016
Meet midwife during labour may look after other women if unit busy	-0.124	-0.281 to 0.032	0.119
**Distance from home** [0 to 15 minutes]	[0]		
15 to 30 minutes	-0.224	-0.447 to -0.001	0.049
30 to 60 minutes	-0.185	-0.433 to 0.063	0.143
More than 60 minutes	-0.297	-0.542 to -0.052	0.018
**Time to see doctor** [0–10 minutes]	[0]		
10–20 minutes	0.067	-0.183 to 0.318	0.598
20–40 minutes	0.097	-0.322 to 0.515	0.651
40–60 minutes	0.036	-0.345 to 0.416	0.855
More than 60 minutes	-0.029	-0.408 to 0.349	0.880
**Can partner stay overnight** [Partner cannot stay overnight]	[0]		
Partner can stay on ward shared by others	0.428	0.223 to 0.633	0.000
Partner can stay in room not shared by others	0.599	0.388 to 0.810	0.000
**Chance of straightforward birth** [50% have straightforward birth]	[0]		
60% have straightforward birth	0.138	-0.030 to 0.306	0.106
70% have straightforward birth	0.201	0.009 to 0.392	0.040
**Safety for baby** [Slightly worse than average]	[0]		
Average	0.744	0.577 to 0.911	0.000
Slightly better than average	1.119	0.892 to 1.347	0.000
**STANDARD DEVIATIONS**			
Variable	Coefficient	Standard error	p-value
**Alternative-specific constants (ASCs)** [Home reference ASC]	[0]		
FMU	0.452	0.169	0.000
AMU	0.004	0.184	0.844
Labour ward	0.999	0.184	0.000
**Reputation** [Poor experience]	[0]		
No previous experience	0.027	0.089	0.193
Good experience	0.288	0.084	0.089
**Continuity of care** [Same midwife throughout]	[0]		
One of a team of midwives throughout	0.339	0.097	0.051
Meet midwife for first time during labour, only look after you	0.234	0.086	0.468
Meet midwife during labour may look after other women if unit busy	0.030	0.080	0.502
**Distance from home** [0 to 15 minutes]	[0]		
15 to 30 minutes	0.008	0.114	0.910
30 to 60 minutes	0.494	0.127	0.008
More than 60 minutes	0.722	0.125	0.000
**Time to see doctor** [0–10 minutes]	[0]		
10–20 minutes	0.192	0.128	0.623
20–40 minutes	0.074	0.214	0.455
40–60 minutes	0.060	0.194	0.620
More than 60 minutes	0.566	0.193	0.000
**Can partner stay overnight** [Partner cannot stay overnight]	[0]		
Partner can stay on ward shared by others	0.142	0.105	0.464
Partner can stay in room not shared by others	0.575	0.107	0.000
**Chance of straightforward birth** [50% have straightforward birth]	[0]		
60% have straightforward birth	0.018	0.086	0.510
70% have straightforward birth	0.616	0.098	0.000
**Safety for baby** [Slightly worse than average]	[0]		
Average	0.036	0.085	0.485
Slightly better than average	0.862	0.116	0.000
**GOODNESS OF FIT**			
**Number of observations**	7710		
**Number of choices**	3855		
**Log likelihood**	-2394		

Statistically significant standard deviations were observed for the ASCs for AMU and OU, two levels in ‘distance from home’ and one level for ‘can partner stay overnight’, ‘chance of straightforward birth’ and ‘safety for baby’ each. This indicated that for these attributes there was considerable random (unexplained) variation across women.

### Post estimation scenario analysis

Our analyses show that predicted probabilities of choosing each setting and assuming participants had access to all four settings (and all else being equal): 33.7% of women might choose OU; 28.5% AMU; 23.3% FMU; and 14.4% home. These figures are shown in Table 4 along with the choices actually reported by women in this study (full results of scenario analyses is available in Appendix E in S1 File).

**Table 4:** Comparison of change in prediction probabilities for each birth setting in response to changes in selected attributes.

		**Home**	**FMU**	**AMU**	**Labour ward**
Actual choice by women (%)		12.5	6.0	19.1	62.3
*Baseline uptake (%)		14.4 (11.0, 18.3)	23.3 (18.4, 28.3)	28.5 (23.9, 34.1)	33.7 (29.8, 38.5)
**Change in probability from baseline [% (95%CI)]					
Attribute	Change				
Safety for baby	[0] **Slightly worse than average** to	11.5 (4.6, 18.3)	14.2 (6.3, 22.1)	16.3 (8.1, 24.5)	15.2 (6.8, 23.4)
	[1] **Average**				
	[0] **Slightly worse than average** to	21.5 (14.2, 28.8)	23.0 (15.0, 31.0)	25.2 (17.0, 33.5)	23.6 (15.2, 31.9)
	[2] **Slightly better than average**				
Chance of straightforward birth	[0] **50% have straightforward birth** to	1.7 (-4.5, 7.9)	2.3 (-5.1, 9.7)	2.7 (-5.2, 10.6)	2.6 (-5.6, 10.8)
	[1] **60% have straightforward birth**				
	[0] **50% have straightforward birth** to	4.5 (-1.9, 11.0)	5.7 (-1.9, 13.3)	5.7 (-2.3, 13.7)	5.0 (-3.3, 13.3)
	[2] **70% have straightforward birth**				
Can partner stay overnight	[0] **No** to		7.9 (0.2, 15.6)	9.2 (1.1, 17.3)	8.8 (0.4, 17.1)
	[1] **On ward shared with others**				
	[0] **No** to		12.5 (4.6, 20.3)	14.3 (6.1, 22.4)	13.4 (5.0, 21.8)
	[2] **In room not shared with others**				
Time to see doctor	[0] **0-10mins** to			1.8 (-6.1, 9.7)	1.3 (-6.9, 9.5)
	[1] **10-20mins**				
	[1] **10-20mins** to	0.4 (-5.7, 6.5)	0.6 (-6.8, 7.9)		
	[2] **20-40mins**				
Distance from home	[0] **0–15 mins** to		-1.6 (-8.8, 5.7)	-0.8 (-8.6, 6.9)	-0.9 (9.0, 7.3)
	[2] **30–60 mins**				
Continuity of care	[0] **You get to know your midwife during your pregnancy and where possible they look after you throughout labour and birth** to	-1.4 (-7.3, 4.6)	-2.1 (-9.3, 5.1)	-2.4 (-10.1, 5.4)	-2.2 (-10.3, 5.9)
	[2] **You meet the midwife for the first time during labour (e.g. on arrival at the unit), and they look after your throughout labour and birth**.				
Reputation	[0] **Your friends/family have had a poor experience of this option** to	5.7 (-0.8, 12.2)	7.3 (-0.3, 15.0)	8.4 (0.4,16.5)	8.0 (-0.4, 16.3)
	[2]**Your friends/family have a good experience of this option**				

* Baseline uptake represents the predicted probabilities that women would choose each setting assuming participants had access to all four settings and all else being equal

** Refers to change from baseline uptake and represents absolute change in proportions

We also investigated the impact of single level changes to attributes (again all else being equal) on predicted uptake, and these results are shown in Table 4. For example, increasing the level of safety for the baby at home from ‘slightly worse than average’ to ‘average’ increased predicted uptake of home birth by 11.5% (95%CI 4.6 to 18.3). Similarly, increasing safety for baby by the same level for an FMU was associated with an increase in predicted uptake of 14.2% (95%CI 6.3 to 22.1). Changes in the attributes ‘safety for the baby’ (i.e. increased safety) and ‘can partner stay overnight’ (i.e. facilities for partner to stay after birth) were associated with larger increases in predicted uptake across settings compared to the remaining attributes. ‘Time to see the doctor’ and ‘distance from home’ were associated with negligible changes in predicted uptake across settings.

## Discussion

### Main findings

We investigated which attributes, or characteristics of care, are important to women when choosing where to give birth. All but one of the attributes included in this DCE were shown to be important in women’s preferences for birth setting. Women preferred midwifery units (MUs) and obstetric units (OUs) to planned home birth, and this mirrored the decisions made by women in this study who had already selected their setting. Our scenario analysis identified safety for the baby, chance of having a straightforward birth, and facilities for the woman’s partner to stay overnight as important drivers of choice.

Participants were generally open to choosing all settings, with OU and midwifery units preferred to home birth. Data from the scenario analysis suggests that if all settings were available to the participants of this study and women were aware of the differences between settings, then the proportion choosing to give birth in OUs could be reduced by as much as 46% (from 62.3% to 33.7%). Almost all of these women would instead choose birth in a MU setting (52%). However, only half of the women in our study reported being aware of having access to an AMU, and a third were aware of the availability of an FMU.

### Strengths and limitations

A strength of DCEs in general is that they present information to participants in a way that resembles “real-world” scenarios; that is to say that decisions are based on a number of different and sometimes competing priorities. Asking participants to make a number of choices between hypothetical scenarios enables us to build up a picture of what is important in decision making, including service attributes which may not currently be available to all. We can only learn so much from asking women about their experiences of birth setting and choice when provision of services and information is variable. For example, even when local provision is poor women may report being satisfied with care because they may not be aware of better alternatives.[28] A recent systematic review and meta-analysis of the external validity of DCEs concluded that they have moderate accuracy when predicting health related choices, and the authors concluded that DCEs can be useful in predicting real-world behaviour.[22]

Developing the attributes and levels for a discrete choice experiment is important to the overall validity of the study, however this is often a “black-box” in reports of DCEs, whereby it is difficult to know how the attributes were identified and developed.[20, 29] A strength of this study is the significant effort that went into developing and refining the attributes using a broad range of evidence and primary research, as well as iterative development through stakeholder and patient and public involvement. A comprehensive list of attributes was included, however it is possible that, for some women, not all attributes of importance were included. DCEs present information in a way that attempts to model how decisions are made in real-life, involving many competing and overlapping factors. Birth choice is a complex topic, and while we present “averaged” findings for women, this may not represent the complexity of choice for individual women.

Recruitment to the study was slow when advertising through social media, therefore a decision was made to engage an online panel to identify and invite potentially eligible women. While this approach helped to recruit more than the initial projected sample size, it does have limitations. Participants in the online panel were incentivised using reward points for completion of the survey, so it may be the case that some participants may have completed the survey without engaging with the questions simply in order to receive the award. While the sample of women in this study matched that of the general population on a number of demographics, the women who participated tended to be better educated, were likely to have had a greater interest in choice of birth setting than the wider population, and may have been more likely to consider planning birth in a MU or at home.

It is worth reiterating that we present data on where pregnant women would like to give birth, along with their stated preferences for birth settings, however we do not know where they went on to give birth, or to what extent this reflected the choices they had already made.

Care should be taken in interpreting the results of this study beyond the included sample. When conducting internet based surveys it is often difficult to calculate a response rate (the proportion of participants who saw the advert and then took part in the study). This is an important factor as it can tell us something about the differences between those who took part and those that did not. While the sample of women in this study matched that of the general population on a number of demographics, the women who participated tended to be better educated, were likely to have had a greater interest in choice of birth setting than the wider population, and were more likely to consider planning birth in a MU or at home. Participation was also limited to being completed on a computer or tablet, therefore excluding those who only had access to a smart phone. Also, a quarter of women reported that they had risk factors that might contraindicate birth outside an OU, which might have therefore limited their actual choices. It is worth emphasising however that we investigated women’s preferences rather than where they actually gave birth. We aim in future studies to investigate how women’s preferences map onto the settings available to them geographically, as well as how this links to where they give birth.

### Interpretation

It has been four years since national guidelines in England were updated to include, for the first time, topics to frame discussions about birth choice with pregnant women.[1] There have been a number of previous studies using stated-preference methods (i.e. discrete choice, conjoint analysis, willingness to pay) to investigate women’s preferences for birth setting or birth experience: three in Scotland[30–32], one in England[33], and one in the Republic of Ireland.[34] However most are not current, with the most recent study in England conducted over 15 years ago, and so further investigation is timely, particularly in light of the changes to national guidelines in 2014. This study investigated some of the same attributes investigated in previous studies, including continuity of care, distance to the unit, and availability of medical staff. However our study also included several attributes that have not been included in other studies, such as reputation, safety for the baby, intervention rate, and the possibility for partner to stay overnight, all of which were shown to be important to women when choosing a birth setting.

We included a number of attributes that may not be amenable to change by service providers, but give important insights into women’s decision-making processes when selecting a birth setting. One explanation for why women at low risk of birth complications still overwhelmingly give birth in OUs is that there may be a misperception that it is safer, due to having health care professionals at hand.[35] Safety for the baby was indeed a key driver of preference in this study. But we also found in our scenario analysis that the chance of having a straightforward birth without intervention was another important factor for women. This is a notable finding. The Birthplace study provided good quality evidence about the relative safety for the baby of each birth setting, showing that for nulliparous and multiparous women, MUs were as safe as OUs, but there is also strong evidence from Birthplace and other studies that planning birth in a MU is associated with significantly increased chances of having a straightforward birth without intervention.[2, 36] This finding suggests that a significant proportion of women might choose birth settings other than hospital OUs if they were aware of the evidence on straightforward birth and convinced that there would be no impact on the safety for their baby. Research has highlighted the need for improvements in midwives’ knowledge about birth settings, as well as the need for pragmatic and understandable birth place discussions that contain standardised content.[37, 38]

It could also be the case that women who choose birth in an OU are not aware of other birth options. It has been shown that women and professionals often assume that birth will take place in the hospital environment.[17, 35] According to a national survey of women’s maternity care experiences in 2014, only a quarter of women (25%) were aware of all four options for place of birth; a further 40% were aware of two or three options; and 33% had one choice only.[8] The findings from our focus group study including 69 women indicated that women gather information about where they plan to give birth from multiple sources, not just their midwife or the health system. So the challenge of impacting on these assumptions is not insignificant.[18]

We have shown in this study that the option for their partner to stay overnight after the birth was important to women in their decision-making about their birth setting. Previous studies have highlighted the importance of partners in decision making, and the need to avoid making partners feel like ‘outsiders’.[39, 40] We focussed on the preferences of pregnant women in this study, but future studies should investigate attributes relating to the woman’s partner, as well exploring the role of partners’ preferences in the decision-making process.

Our modelling exercise demonstrated that there was significant random, or unexplained variation in women’s preferences across some attributes and levels indicating that there are subgroups of women with distinct patterns of preferences. Future work should investigate whether the random heterogeneity observed in this study leads to specific patterns of preferences that can be mapped onto available birth settings.

## Conclusion

This study investigated women’s preferences for birth setting and found a number of factors that are important to women, particularly ‘safety for the baby’, ‘chance of having a straightforward birth’ and ‘can the woman’s partner stay overnight following birth’. If all birth settings were available for women, and they were fully informed about the benefits of each of them, it is likely that more low risk women currently giving birth in OUs would choose to plan birth in a midwifery unit.

## Supporting information

S1 File Appendix A. Experimental design. Appendix B. Sample size calculation. Appendix C. Screenshot of DCE question as it appeared on-line. Appendix D. Information about birth setting. Appendix E. Scenario analyses. Table A. Where did women find information about informed birth choice. Table B. Most and least important attributes in preferences.
(DOCX)
