# Supplementary material for: Exploring women’s preferences for birth settings in England: A discrete choice experiment
Source: PLoS One. 2019 Apr 11;14(4):e0215098. doi: 10.1371/journal.pone.0215098 (PMC6459528; doi:10.1371/journal.pone.0215098)
Supplement: S1 File — Appendix A. Experimental design. Appendix B. Sample size calculation. Appendix C. Screenshot of DCE question as it appeared on-line. Appendix D. Information about birth setting. Appendix E. Scenario analyses. Table A. Where did women find information about informed birth choice. Table B. Most and least important attributes in preferences. (DOCX) [file pone.0215098.s001.docx]

**SUPPORTING INFORMATION (S1)**

**Exploring women’s preferences for birth settings in England: a discrete choice experiment**

Authors: Fletcher BR^1,2^, Rowe R^1^, Hollowell J^1^, Scanlon M^3^, Hinton L^2^, Rivero-Arias O^1^*

^1^National Perinatal Epidemiology Unit, Nuffield Department of Population Health, University of Oxford, UK

^2^Nuffield Department of Primary Care Health Sciences, University of Oxford, UK

^3^BirthChoiceUK, London, UK

*Corresponding author: Dr Oliver Rivero-Arias, Associate Professor, National Perinatal Epidemiology Unit, University of Oxford, Old Road Campus, Headington, Oxford, UK. Tel: +44 (0) 1865 617908. Email: oliver.rivero@npeu.ox.ac.uk

**Supplementary information**

**Appendix 1** Information about birth settings……………………………………………………………..……………..**Page 2**

**Appendix 2** Experimental design (NGene syntax, restrictions and final design……….……………..….**Page 3**

**Appendix 3** Screen shot of DCE question as it appeared on-line…………….…………………..……………..**Page 8**

**Appendix 4** Sample size calculation…………………………………………………………………………………...………**Page 9**

**Table 1** Most and least important attributes in preferences………………………………….……………..….**Page 10**

**Table 2** Where did you find information that helped your choice of birth setting?.....................**Page 10**

**Appendix 5** Scenario analyses…………………………………………………………………….……….….…………..….**Page 11**

**APPENDIX 1: INFORMATION ABOUT BIRTH SETTINGS PROVIDED IN INFORMATIONAL VIDEO**

Currently, women with straightforward pregnancies should be offered a choice of four birth settings – a hospital ‘labour ward’, two types of midwifery unit or a home birth. Here is a bit more about each one

At a labour ward in a hospital, you would usually be looked after by a midwife while having your baby but there are doctors and medical care on hand if you need them.

In midwifery units, also called birth centres, midwives provide all the care to women giving birth. These tend to have a more homely environment that feels less like a hospital. There are two types of midwifery unit:

Alongside midwifery units are in hospitals but are separate from labour wards so you would need to transfer to a different part of the hospital if you needed to see a doctor during labour or shortly after birth.

Freestanding midwifery units are on a separate site from the nearest main hospital, where there are no doctors or specialist medical care. If you need to see a doctor you would have to transfer to hospital, by car or ambulance.

If you have a home birth a midwife will look after you while you have your baby. Again, if you need to see a doctor you would have to transfer to hospital, by car or ambulance.

In all the settings, you can use natural methods of pain relief such as relaxation, massage and moving around to get comfortable. You can also use a birthing pool if there is one, and some medical pain relief such as gas and air or pethidine may also be available.

But epidural pain relief is only available in a labour ward.

**APPENDIX 2: EXPERIMENTAL DESIGN**

**1.1 Attributes and levels and restrictions to design**

**Attribute A (setting)**

**0** Home

**1** FMU

**2** AMU

**3** OU

**Attribute B (reputation)**

**0** Poor experience

**1** No previous experience

**2** Good experience

**Attribute C (Continuity of care)**

**0** You get to know your midwife during your pregnancy and where possible they look after you throughout labour and birth.

**1** You meet a team of 4-6 midwives during pregnancy, one of whom looks after you throughout labour and birth.

**2** You meet the midwife for the first time during labour (e.g. on arrival at the unit), and they look after your throughout labour and birth.

**3** You meet a midwife for the first time during labour. They will look after you during labour and birth, but if the unit is busy they may look after other women in labour at the same time.

|  | **Home** | **FMU** | **AMU** | **OU** |
| --- | --- | --- | --- | --- |
| **0** | X | X | X | X |
| **1** | X | X | X | X |
| **2** | X | X | X | X |
| **3** |  | X | X | X |

**Attribute D (Distance from home)**

**0** 0 to 15 minutes

**1** 15 to 30 minutes

**2** 30 to 60 minutes

**3** More than 60 minutes

|  | **Home** | **FMU** | **AMU** | **OU** |
| --- | --- | --- | --- | --- |
| **0** | X | X | X | X |
| **1** |  | X | X | X |
| **2** |  | X | X | X |
| **3** |  | X | X | X |

**Attribute E (Time to see doctor)**

**0** 0 to 10 minutes

**1** 10 to 20 minutes

**2** 20 to 40 minutes

**3** 40 to 60 minutes

**4** More than 60 minutes

|  | **Home** | **FMU** | **AMU** | **OU** |
| --- | --- | --- | --- | --- |
| **0** |  |  | X | X |
| **1** | X | X | X | X |
| **2** | X | X |  |  |
| **3** | X | X |  |  |
| **4** | X | X |  |  |

**Attribute F (Can your partner stay overnight after the birth of your baby?)**

**0** Your partner cannot stay with you overnight

**1** Your partner can stay with you overnight on a postnatal ward shared with others

**2** Your partner can stay with you overnight in a room not shared with others

|  | **Home** | **FMU** | **AMU** | **OU** |
| --- | --- | --- | --- | --- |
| **0** |  | X | X | X |
| **1** |  | X | X | X |
| **2** | X | X | X | X |

**Attribute G (Chance of straightforward birth)**

**0** 5 out of 10 women have straightforward birth, 5 out of 10 have intervention

**1** 6 out of 10 women have straightforward birth, 4 out of 10 have intervention

**2 7** out of 10 women have straightforward birth, 3 out of 10 have intervention

**Attribute H (Safety for baby)**

**0** Slightly worse than average (10 of 1,000 have a poor outcome for baby, 990 of 1,000 births baby is born healthy)

**1** Average (4 of 1,000 births have poor outcome for baby, 996 of 1,000 births baby is born healthy)

**2** Slightly better than average (2 of 1,000 births have a poor outcome for baby, 998 of 1,000 births baby is born healthy)

**1.2 Experimental design syntax (NGene)**

Design

;alts = alt1, alt2

;rows = 60

;eff = (mnl, d)

;cond:

If(alt1.A = 0, alt1.C = [0,1,2]),

If(alt2.A = 0, alt2.C = [0,1,2]),

If(alt1.A = 0, alt1.D = 0),

If(alt2.A = 0, alt2.D = 0),

If(alt1.A = [1,2,3], alt1.D = [0,1,2,3]),

If(alt2.A = [1,2,3], alt2.D = [0,1,2,3]),

If(alt1.A = [0,1], alt1.E = [1,2,3,4]),

If(alt2.A = [0,1], alt2.E = [1,2,3,4]),

If(alt1.A = [2,3], alt1.E = [0,1]),

If(alt2.A = [2,3], alt2.E = [0,1]),

If(alt1.A = [1,2,3], alt1.F = [0,1,2]),

If(alt2.A = [1,2,3], alt2.F = [0,1,2]),

If(alt1.A = 0, alt1.F = 2),

If(alt2.A = 0, alt2.F = 2)

;block=4

;model:

U(alt1) = b1 + b2 * A[0,1,2,3] + b3 * B[0,1,2] + b4 * C[0,1,2,3] + b5 * D[0,1,2,3] + b6 * E[0,1,2,3,4] + b7 * F[0,1,2] + b8 * G[0,1,2] + b9 * H[0,1,2] /

U(alt2) = b2 * A[0,1,2,3] + b3 * B[0,1,2] + b4 * C[0,1,2,3] + b5 * D[0,1,2,3] + b6 * E[0,1,2,3,4] + b7 * F[0,1,2] + b8 * G[0,1,2] + b9 * H[0,1,2]

$

Syntax saved as – Final design for pilot BF25072017.ngs

Design saved as – Final design for pilot BF25072017.ngd

Evaluation number = 175

D-efficiency = 96.9%

**1.3 Level balance**

**Overall**

| **Attribute** | **Level** | | | | |
| --- | --- | --- | --- | --- | --- |
|  | **0** | **1** | **2** | **3** | **4** |
| **A** | 23 | 53 | 22 | 22 |  |
| **B** | 40 | 40 | 40 |  |  |
| **C** | 34 | 32 | 25 | 29 |  |
| **D** | 44 | 28 | 24 | 24 |  |
| **E** | 25 | 28 | 22 | 22 | 23 |
| **F** | 35 | 30 | 55 |  |  |
| **G** | 40 | 40 | 40 |  |  |
| **H** | 40 | 40 | 40 |  |  |

**By block**

| **Attribute A** | **Block 1** | **Block 2** | **Block 3** | **Block 4** |
| --- | --- | --- | --- | --- |
| **0** | **6** | **6** | **6** | **5** |
| **1** | **14** | **12** | **12** | **15** |
| **2** | **5** | **3** | **9** | **5** |
| **3** | **5** | **9** | **3** | **5** |

| **Attribute B** | **Block 1** | **Block 2** | **Block 3** | **Block 4** |
| --- | --- | --- | --- | --- |
| **0** | **9** | **15** | **6** | **10** |
| **1** | **12** | **7** | **10** | **11** |
| **2** | **9** | **8** | **14** | **9** |

| **Attribute C** | **Block 1** | **Block 2** | **Block 3** | **Block 4** |
| --- | --- | --- | --- | --- |
| **0** | **6** | **8** | **9** | **11** |
| **1** | **10** | **8** | **8** | **6** |
| **2** | **7** | **6** | **5** | **7** |
| **3** | **7** | **8** | **8** | **6** |

| **Attribute D** | **Block 1** | **Block 2** | **Block 3** | **Block 4** |
| --- | --- | --- | --- | --- |
| **0** | **12** | **11** | **11** | **10** |
| **1** | **5** | **8** | **10** | **5** |
| **2** | **6** | **3** | **5** | **10** |
| **3** | **7** | **8** | **4** | **5** |

| **Attribute E** | **Block 1** | **Block 2** | **Block 3** | **Block 4** |
| --- | --- | --- | --- | --- |
| **0** | **6** | **7** | **8** | **4** |
| **1** | **7** | **7** | **7** | **7** |
| **2** | **6** | **2** | **6** | **8** |
| **3** | **6** | **7** | **3** | **6** |
| **4** | **5** | **7** | **6** | **5** |

| **Attribute F** | **Block 1** | **Block 2** | **Block 3** | **Block 4** |
| --- | --- | --- | --- | --- |
| **0** | **8** | **8** | **10** | **9** |
| **1** | **9** | **8** | **8** | **5** |
| **2** | **13** | **14** | **12** | **16** |

| **Attribute G** | **Block 1** | **Block 2** | **Block 3** | **Block 4** |
| --- | --- | --- | --- | --- |
| **0** | **10** | **8** | **13** | **9** |
| **1** | **9** | **14** | **8** | **9** |
| **2** | **11** | **8** | **9** | **12** |

| **Attribute H** | **Block 1** | **Block 2** | **Block 3** | **Block 4** |
| --- | --- | --- | --- | --- |
| **0** | **10** | **8** | **10** | **11** |
| **1** | **12** | **7** | **9** | **12** |
| **2** | **8** | **14** | **11** | **7** |

**APPENDIX 3: SCREENSHOT OF DCE QUESTION AS IT APPEARED ON LIMESURVEY**


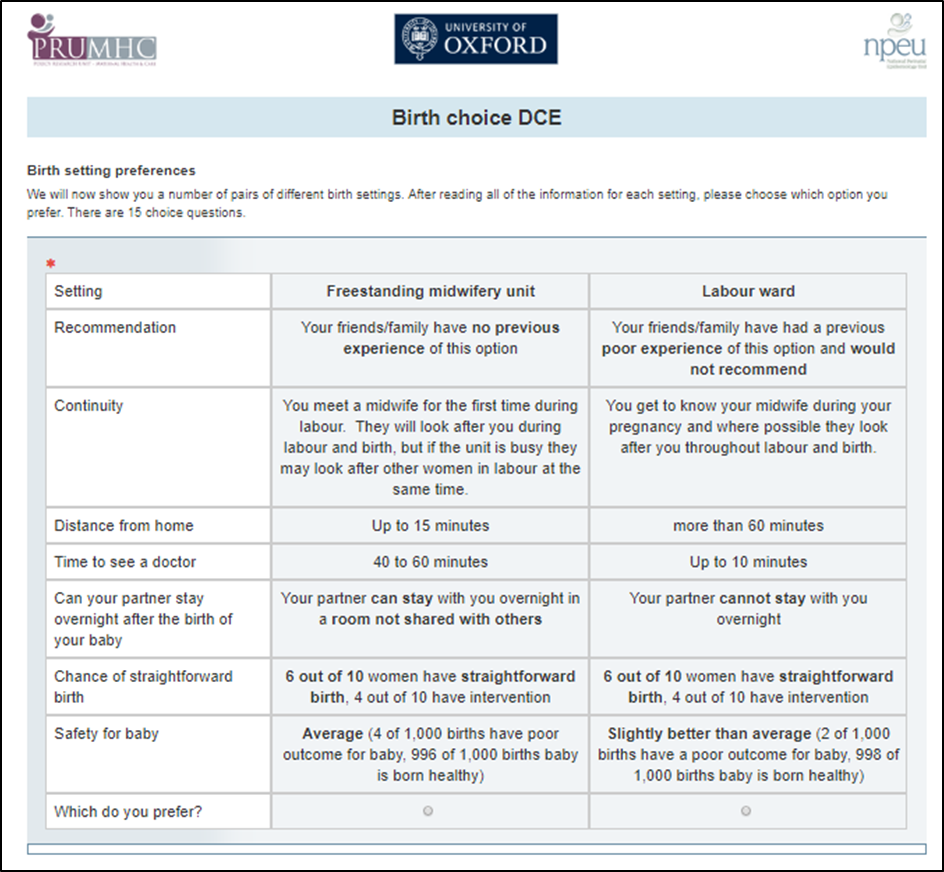


**APPENDIX 4: SAMPLE SIZE CALCULATION**

There is currently no definitive method of calculating sample sizes for DCEs, and this is a developing field.(de Bekker-Grob 2015) Johnson and Orme proposed the following “rule of thumb” sample size calculation for DCEs (Johnson 2003):

*nta/c* ≥ 500

Where *n* is the number of respondents, *t* is the number of tasks, *a* is the number of alternatives per task, and when considering main effects *c* is equal to the largest number of levels of any of the attributes. 500 relates to the number of times each main effect is represented across the design, and it has been argued that this should be a minimum threshold, and that it would be better to have 1,000. For this study to meet the lower threshold, 84 participants would be required, and for the upper threshold, 168 would be needed. We therefore aimed to sample at least 200 women.

**TABLE 1: MOST/LEAST IMPORTANT ATTRIBUTES IN PREFERENCES**

*Participants were asked to choose the three most and three least important attributes when making choices in the questionnaire

|  | **Most important**  **N=257** | **Least important**  **N=257** |
| --- | --- | --- |
| Setting | 61 (**23.7%**; 95%CI 19.0 to 29.3) | 108 (**42.0%**; 95%CI 36.2 to 48.1) |
| Reputation | 117 (**45.5%**; 95%CI 39.6 to 45.5) | 114 (**44.4%**; 95%CI 38.4 to 50.5) |
| Continuity of care | 101 (**39.3%**; 95%CI 33.5 to 45.4) | 107 (**41.6%**; 95%CI 35.8 to 47.8) |
| Distance from home | 83 (**32.3%**; 95%CI 26.9 to 38.2) | 123 (**47.9%**; 95%CI 41.8 to 54.0) |
| Time to see a doctor | 111 (**43.2%**; 95%CI 37.3 to 49.3) | 91 (**35.4%**; 95%CI 29.8 to 41.4) |
| Partner able to stay overnight | 84 (**32.7%**; 95%CI 27.2 to 38.6) | 110 (**42.8%**; 95%CI 36.9 to 48.9) |
| Chance of straightforward birth | 95 (**37.0%**; 95%CI 31.2 to 43.0) | 61 (**23.7%**; 95%CI 19.0 to 29.3) |
| Safety for baby | 119 (**46.3%**; 95%CI 40.3 to 52.4) | 57 (**22.2%**; 95%CI 17.5 to 27.7) |

**TABLE 2: WHERE DID YOU FIND INFORMATION THAT HELPED YOUR CHOICE OF BIRTH SETTING?**

|  | **N=257** |
| --- | --- |
| Online | 124 (48%) |
| Midwife | 176 (68%) |
| GP | 101 (39%) |
| Obstetrician | 42 (16%) |
| Family/friends | 88 (34%) |
| App store | 17 (7%) |
| App recommended by doctor/midwife | 8 (3%) |
| Other* | 19 (7%) |

*Included: hypnobirthing book/course, own research/knowledge and experience, magazines, “didn’t feel had a choice”

**APPENDIX 5: SCENARIO ANALYSES**

**Safety for baby**

|  | Home | FMU | AMU | Labour ward |
| --- | --- | --- | --- | --- |
|  | Probability (%) | Probability (%) | Probability (%) | Probability (%) |
| Baseline | 14.4  (11.0, 18.3) | 23.3  (18.4, 28.3) | 28.5  (23.9, 34.1) | 33.7  (29.8, 38.5) |
| 1 Increase from “slightly worse than average” to “average” for home | 11.5  (4.6, 18.3) | -3.2  (-10.3, 4.0) | -4.2  (-11.8, 3.4) | -4.1  (-12.1, 3.94) |
| 2 Increase from “slightly worse than average” to “slightly better than average” for home | 21.5  (14.2, 28.8) | -5.8  (-12.8, 1.1) | -7.5  (-15.0, -0.1) | -8.1  (-16.0, -0.3) |
| 3 Increase from “slightly worse than average” to “average” for FMU | -2.9  (-8.7, 2.9) | 14.2  (6.3, 22.1) | -5.6  (-13.2, 2.3) | -5.7  (-13.7, 2.3) |
| 4 Increase from “slightly worse than average” to “slightly better than average” for FMU | -4.4  (-10.0, 1.3) | 23.0  (15.0, 31.0) | -8.7  (-16.0, -1.3) | -9.9  (-17.7, -2.2) |
| 5 Increase from “slightly worse than average” to “average” for AMU | -3.7  (-9.4, 2.0) | -5.5  (-12.5, 1.5) | 16.3  (8.1, 24.5) | -7.2  (-15.1, 0.7) |
| 6 Increase from “slightly worse than average” to “slightly better than average” for AMU | -5.4  (-10.9, 0.2) | -8.2  (-14.9, -1.4) | 25.2  (17.0, 33.5) | -11.7  (-19.4, -4.0) |
| 7 Increase from “slightly worse than average” to “average” for labour ward | -3.4  (-9.1, 2.3) | -5.2  (-12.2, 1.8) | -6.7  (-14.2, 0.8) | 15.2  (6.8, 23.4) |
| 8 Increase from “slightly worse than average” to “slightly better than average” for labour ward | -5.2  (-10.8, 0.4) | -8.1  (-14.9, -1.3) | -10.3  (-17.5, -3.0) | 23.6  (15.2, 31.9) |

**Chance of straightforward birth**

|  | Home | FMU | AMU | Labour ward |
| --- | --- | --- | --- | --- |
|  | Probability (%) | Probability (%) | Probability (%) | Probability (%) |
| Baseline | 14.4  (11.0, 18.3) | 23.3  (18.4, 28.3) | 28.5  (23.9, 34.1) | 33.7  (29.8, 38.5) |
| 9 Increase from “50%” to “60%” for home | 1.7  (-4.5, 7.9) | -0.5  (-7.8, 6.8) | -0.6  (-8.4, 7.2) | -0.6  (-8.8, 7.6) |
| 10 Increase from “50%” to “70%” for home | 4.5  (-1.9, 11.0) | -1.6  (-8.8, 5.6) | -1.4  (-9.2, 6.3) | -1.5  (-9.7, 6.6) |
| 11 Increase from “50%” to “60%” for FMU | -0.5  (-6.5, 5.6) | 2.3  (-5.1, 9.7) | -0.9  (-8.7, 6.8) | -0.9  (-9.1, 7.2) |
| 12 Increase from “50%” to “70%” for FMU | -1.2  (-7.2, 4.7) | 5.7  (-1.9, 13.3) | -2.4  (-10.1, 5.3) | -2.0  (-10.1, 6.1) |
| 13 Increase from “50%” to “60%” for AMU | -0.6  (-6.6, 5.4) | -0.9  (-8.2, 6.3) | 2.7  (-5.2, 10.6) | -1.2  (-9.3, 7.0) |
| 14 Increase from “50%” to “70%” for AMU | -1.0  (-6.9, 5.0) | -2.4  (-9.6, 4.7) | 5.7  (-2.3, 13.7) | -2.3  (-10.4, 5.8) |
| 15 Increase from “50%” to “60%” for labour ward | -0.6  (-6.6, 5.4) | -0.9  (-8.2, 6.4) | -1.1  (-5.6, 10.8) | 2.6  (-5.6, 10.8) |
| 16 Increase from “50%” to “70%” for labour ward | -1.1  (-7.0, 4.9) | -1.8  (-9.0, 5.4) | -2.1  (-9.8, 5.6) | 5.0  (-3.3, 13.3) |

**Can partner stay overnight**

|  | Home | FMU | AMU | Labour ward |
| --- | --- | --- | --- | --- |
|  | Probability (%) | Probability (%) | Probability (%) | Probability (%) |
| Baseline | 14.4  (11.0, 18.3) | 23.3  (18.4, 28.3) | 28.5  (23.9, 34.1) | 33.7  (29.8, 38.5) |
| 17 Change from “no” to “on ward” at FMU | -1.6  (-7.5, 4.3) | 7.9  (0.2, 15.6) | -3.1  (-10.8, 4.5) | -3.1  (-11.2, 4.9) |
| 18 Change from “no” to “in private room” at FMU | -2.5  (-8.3, 3.4) | 12.5  (4.6, 20.3) | -4.9  (-12.5, 2.7) | -5.0  (-13.0, 2.9) |
| 19 Change from “no” to “on ward” at AMU | -2.1  (-8.0, 3.7) | -3.1  (-10.2, 4.1) | 9.2  (1.1, 17.3) | -4.0  (-12.1, 4.0) |
| 20 Change from “no” to “in private room” at AMU | -3.1  (-8.9, 2.6) | -4.8  (-11.8, 2.2) | 14.3  (6.1, 22.4) | -6.3  (-14.2, 1.6) |
| 21 Change from “no” to “on ward” at labour ward | -2.0  (-7.8, 3.9) | -2.9  (-10.1, 4.2) | -3.9  (-11.5, 3.8) | 8.8  (0.4, 17.1) |
| 22 Change from “no” to “in private room” at labour ward | -3.0  (-8.8, 2.8) | -4.6  (-11.6, 2.4) | -5.8  (-13.4, 1.7) | 13.4  (5.0, 21.8) |

**Time to see doctor**

|  | Home | FMU | AMU | Labour ward |
| --- | --- | --- | --- | --- |
|  | Probability (%) | Probability (%) | Probability (%) | Probability (%) |
| Baseline | 14.4  (11.0, 18.3) | 23.3  (18.4, 28.3) | 28.5  (23.9, 34.1) | 33.7  (29.8, 38.5) |
| 23 Increase from “10-20 mins” to “20-40 mins” at home | 0.4  (-5.7, 6.5) | -0.1  (-7.3, 7.2) | -0.1  (-7.9, 7.7) | -0.2  (-8.4, 8.0) |
| 24 Increase from “10-20 mins” to “20-40 mins” at FMU | -0.1  (-6.1, 6.0) | 0.6  (-6.8, 7.9) | -0.1  (-7.9, 7.7) | -0.4  (-8.6, 7.7) |
| 25 Increase from “0-10 mins” to “10-20 mins” at AMU | -0.4  (-6.5, 5.6) | -0.7  (-7.8, 6.6) | 1.8  (-6.1, 9.7) | -0.6  (-8.8, 7.5) |
| 26 Increase from “0-10 mins” to “10-20 mins” at labour ward | -0.3  (-6.3, 5.7) | -0.4  (-7.7, 6.9) | -0.6  (-8.4, 7.2) | 1.3  (-6.9, 9.5) |

**Distance from home**

|  | Home | FMU | AMU | Labour ward |
| --- | --- | --- | --- | --- |
|  | Probability (%) | Probability (%) | Probability (%) | Probability (%) |
| Baseline | 14.4  (11.0, 18.3) | 23.3  (18.4, 28.3) | 28.5  (23.9, 34.1) | 33.7  (29.8, 38.5) |
| 27 Increase from “0-15 mins” to “30-60 mins” at FMU | 0.4  (-5.7, 6.5) | -1.6  (-8.8, 5.7) | 0.8  (-7.0, 8.7) | 0.3  (-7.8, 8.5) |
| 28 Increase from “0-15 mins” to “30-60 mins” at AMU | 0.1  (-6.0, 6.2) | 0.8  (-6.6, 8.1) | -0.8  (-8.6, 6.9) | -0.1  (-8.2, 8.1) |
| 29 Increase from “0-15 mins” to “30-60 mins” at labour ward | 0.1  (-6.0, 3.2) | 0.5  (-6.8, 7.8) | 0.2  (-7.6, 8.1) | -0.9  (9.0, 7.3) |

**Continuity of care**

|  | Home | FMU | AMU | Labour ward |
| --- | --- | --- | --- | --- |
|  | Probability (%) | Probability (%) | Probability (%) | Probability (%) |
| Baseline | 14.4  (11.0, 18.3) | 23.3  (18.4, 28.3) | 28.5  (23.9, 34.1) | 33.7  (29.8, 38.5) |
| 30 Change from “same midwife pregnancy and birth” to “meet midwife in labour” at home | -1.4  (-7.3, 4.6) | 0.4  (-6.9, 7.7) | 0.5  (-7.3, 8.4) | 0.4  (-7.7, 8.6) |
| 31 Change from “same midwife pregnancy and birth” to “meet midwife in labour” at FMU | 0.4  (-5.7, 6.6) | -2.1  (-9.3, 5.1) | 0.9  (-7.0, 8.7) | 0.8  (-7.4, 9.0) |
| 32 Change from “same midwife pregnancy and birth” to “meet midwife in labour” at AMU | 0.6  (-5.6, 6.7) | 0.9  (-6.5, 8.2) | -2.4  (-10.1, 5.4) | 0.9  (-7.3, 9.1) |
| 33 Change from “same midwife pregnancy and birth” to “meet midwife in labour” at labour ward | 0.5  (-5.6, 6.6) | 0.8  (-6.5, 8.2) | 1.0  (-6.9, 8.8) | -2.2  (-10.3, 5.9) |

**Reputation**

|  | Home | FMU | AMU | Labour ward |
| --- | --- | --- | --- | --- |
|  | Probability (%) | Probability (%) | Probability (%) | Probability (%) |
| Baseline | 14.4  (11.0, 18.3) | 23.3  (18.4, 28.3) | 28.5  (23.9, 34.1) | 33.7  (29.8, 38.5) |
| 34 Change from “poor” to “good” at home | 5.7  (-0.8, 12.2) | -1.6  (-8.8, 5.6) | -2.0  (-9.8, 5.7) | -2.1  (-10.2, 6.1) |
| 35 Change from “poor” to “good” at FMU | -1.5  (-7.4, 4.5) | 7.3  (-0.3, 15.0) | -2.9  (-10.6, 4.8) | -3.0  (-11.0, 5.1) |
| 36 Change from “poor” to “good” at AMU | -1.9  (-7.8, 4.1) | -2.9  (-10.0, 4.3) | 8.4  (0.4, 16.5) | -3.7  (-11.8, 4.3) |
| 37 Change from “poor” to “good” at labour ward | -1.8  (-7.7, 4.2) | -2.8  (-9.9, 4.4) | -3.5  (-11.1, 4.2) | 8.0  (-0.4, 16.3) |
